# Landscape Epidemiology of Tularemia Outbreaks in Sweden

**DOI:** 10.3201/eid1512.090487

**Published:** 2009-12

**Authors:** Kerstin Svensson, Erik Bäck, Henrik Eliasson, Lennart Berglund, Malin Granberg, Linda Karlsson, Pär Larsson, Mats Forsman, Anders Johansson

**Affiliations:** Swedish Defense Research Agency, Umeå, Sweden (K. Svensson, M. Granberg, L. Karlsson, P. Larsson, M. Forsman, A. Johansson); Umeå University, Umeå (K. Svensson, A. Johansson); Örebro University Hospital, Örebro, Sweden (E. Bäck, H. Eliasson); Ljusdal Healthcare Centre, Ljusdal, Sweden (L. Berglund); Umeå University Hospital, Umeå (A. Johansson)

**Keywords:** Francisella tularensis, tularemia, epidemiology, outbreak, bacterial typing, VNTR, SNPs, bacteria, Sweden, research

## Abstract

Summer outbreaks of tularemia that occurred from 1995 through 2005 in 2 locations in Sweden affected 441 persons. We performed an epidemiologic investigation of these outbreaks using a novel strategy, involving high-resolution genotyping of *Francisella tularensis* isolates obtained from 136 patients (using 18 genetic markers developed from 6 *F. tularensis* genome sequences) and interviews with the patients. Strong spatial associations were found between *F. tularensis* subpopulations and the places of disease transmission; infection by some subpopulations occurred within areas as small as 2 km^2^, indicating unidentified environmental point sources of tularemia. In both locations, disease clusters were associated with recreational areas beside water, and genetic subpopulations were present throughout the tularemia season and persisted over years. High-resolution genotyping in combination with patients’ statements about geographic places of disease transmission provided valuable indications of likely sources of infection and the causal genotypes during these tularemia outbreaks.

## Abstract 2

Transmission sites of specific *Francisella tularensis* genotypes were highly localized during natural outbreaks of human tularemia.

Traditional objectives of investigations of infectious disease outbreaks are to identify ways to control ongoing outbreaks and to prevent future outbreaks. However, a paucity of epidemiologic and ecologic knowledge hampers the investigation of tularemia outbreaks caused by the intracellular bacterium *Francisella tularensis*, although it is one of the most virulent pathogens known. The Centers for Disease Control and Prevention lists this pathogen as one of the most potentially dangerous bioterrorism bacteria (*1*). Little is known about natural reservoirs of tularemia, *F. tularensis* transmission mechanisms to humans, and factors influencing the often irregular pattern of outbreaks. Because tularemia is a zoonosis and little ecologic information exists about the causal organism, its prevention and control may require the development of novel outbreak investigation strategies.

In nature, *F. tularensis* is associated with an extremely wide range of hosts and arthropod vectors; a recent review listed 304 susceptible species (*2*). Transmission to humans may occur through a number of routes; skin inoculation by blood-feeding arthropod vectors is one of the most common routes (*3*). The infectious dose can be as low as 10 bacterial cells (*4*). Human tularemia naturally occurs only in biotopes in the Northern Hemisphere. We describe an investigation of a large number of *F. tularensis* isolates from humans. Patients were infected mainly from mosquito bites and had an influenza-like illness, a primary skin ulcer, and enlargement of lymph nodes, the ulceroglandular form of tularemia (*5**,**6*). Tularemia is endemic in Sweden, with seasonal outbreaks and a patchy geographic distribution. The number of infected humans ranged from 27 to 698 per year from 1998 through 2007 in a population of ≈9.1 million (annual incidence rate 0.30–7.78/100,000 persons) (*7*). For comparison, 20–64 humans were reported with tularemia from 2000 through 2006 in the tularemia-endemic US states of Arkansas and Missouri, from a population of ≈8.3 million (annual incidence rate 0.23–0.76/100,000 persons) (*8*). *F. tularensis* subsp. *holarctica* causes tularemia all over the Northern Hemisphere. This is a severe febrile disease but does not generally result in death. A more virulent variety, *F. tularensis* subsp. *tularensis*, exists in North America. It was associated with a human mortality rate of 5%–15% before the advent of effective antimicrobial drug treatments (*4*). *F. tularensis* has a clonal genetic structure, a property that should facilitate tracking the spread of tularemia by genotyping (*9**,**10*).

We demonstrate a strategy to enhance epidemiologic investigations of tularemia by combining geographic data collected from patient interviews and high-resolution genotyping of *F. tularensis* subsp. *holarctica* isolates recovered from tularemia patients. We found that geographic distributions of specific *F. tularensis* subsp. *holarctica* subpopulations were highly localized during outbreaks (infections by some genotypes were restricted to areas as small as 2 km^2^), indicating distinct point sources of infection.

## Materials and Methods

### Study Locations

**Figure 1:**
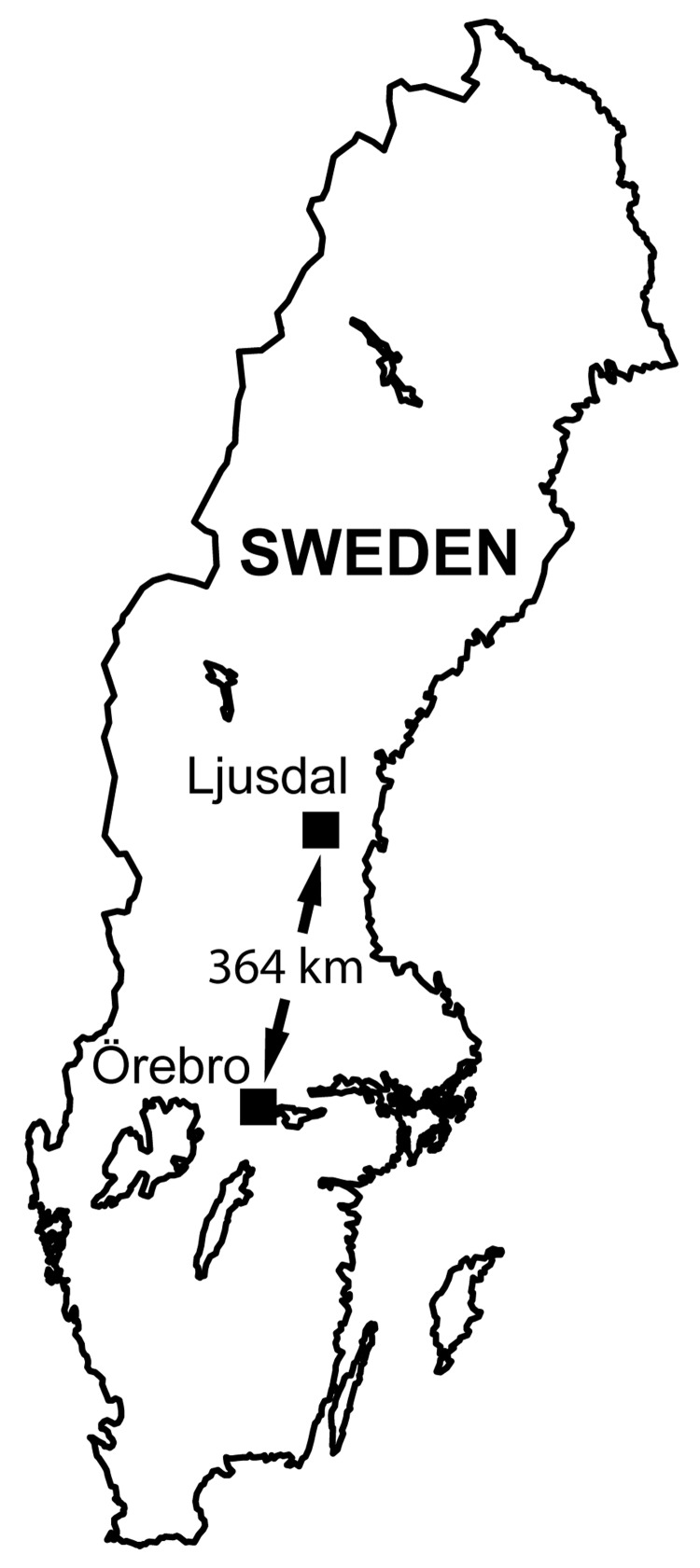
Locations of the 2 tularemia outbreak areas in Sweden, showing Ljusdal and Örebro 364 km apart.

We studied tularemia, which must be reported under Swedish law, in 2 locations 364 km apart: the Municipality of Ljusdal and the County of Örebro (19,384 and 273,956 inhabitants, respectively, in 2005) (*11*) (Figure 1). The human tularemia incidence rates in Ljusdal and Örebro cited here were based on the annual number of human tularemia cases reported to the County Medical Officer for Communicable Diseases. Tularemia has been endemic for several decades in Ljusdal with repeated outbreaks (*12*), whereas in Örebro, incidence of tularemia has been low since the disease was first reported in Sweden in the 1930s (*5*). From 1990 through 1999, Örebro reported only 8 cases.

### Study Period, Isolate Information, and Preparation of DNA

From 1995 through 2005, clinicians in Ljusdal and Örebro sent patient specimens for tularemia serologic analysis, *F. tularensis* culture, and PCR detection to the laboratories at the Swedish Defense Research Agency, Umeå, Sweden; Umeå University Hospital, Umeå; or Örebro University Hospital, Örebro. Culture and PCR diagnostics of ulcer specimens were performed as described elsewhere (*13*). Blood culture was performed by using the instrumented BD Bactec Plus system (Becton Dickinson, Franklin Lakes, NJ, USA). All *Francisella* culture work was performed under BioSafety level 3 laboratory conditions. A tube agglutination test or an ELISA measuring immunoglobulin (Ig) M and IgG was used for serologic analysis, as previously described (*14*). For DNA preparation, *F. tularensis* isolates were recultured, then a loopful of bacteria was suspended in phosphate-buffered saline and heat-killed, and a chaotropic salt method was applied (*6*).

### Identification and Selection of Markers

We used 3 types of genetic markers to provide high-typing resolution and robust categorization of *F. tularensis* subsp. *holarctica* into genetic subpopulations. As described elsewhere, we previously identified 280 insertion/deletion (INDEL) and variable number of tandem repeat (VNTR) markers by multiple alignments of the *Francisella* genomes U112, FSC147, SCHU S4, OSU18, and LVS (*15*). For the current study, we selected 20 of these 280 markers (17 INDELs and 3 VNTRs) that were polymorphic among *F. tularensis* subsp. *holarctica* isolates from Europe and North America. We then added 11 VNTR markers showing polymorphism among subsp. *holarctica* isolates of worldwide origin, and 1 INDEL (Ft-M19) shown to be specific to subsp. *holarctica* (*9*). Finally, we added 2 single nucleotide polymorphism (SNP) markers identified in a comparative BLAST analysis (http://blast.ncbi.nlm.nih.gov/Blast.cgi) of *F. tularensis* subsp. *holarctica* genomes of a worldwide origin including FSC200 (human, Ljusdal, 1998), LVS, RC530, FSC022, FTA, OR96-0246, and OSU18. The 2 SNP markers were selected to be phylogenetically informative while lacking homoplasy among 67 *Francisella* strains of diverse origin. Collectively, the selection process yielded a set of 34 genetic markers that were applied to 48 of the 136 study isolates (24 each from Örebro and Ljusdal). Markers found to be polymorphic among the 48 selected isolates were finally used to genotype all 136 isolates.

### DNA Fragment Analysis and Real-time PCR SNP Assay

DNA fragment analysis of INDELs and VNTRs were performed on a Genetic Analysis System CEQ 8800 (Beckman Coulter Inc., Fullerton, CA, USA) machine, as previously described (*15*). Genomic positions for SNPs and their corresponding PCR amplification primers are presented in Table 1. The SNP states were determined by real-time PCR by using a set of 2 forward primers with different single nucleotide extensions at the 3′ end, and a common reverse primer (*16*). We could read the SNP state by a differential PCR amplification efficiency.

**Table 1:** Attributes of 7 novel typing markers for *Francisella tularensis* subsp. holarctica*

Marker category	Marker name	Genomic position†	Size, bp	Forward primer sequence (5′ →3′)	Reverse primer sequence (5′ → 3′)
INDEL	Ftind39	798173-4	11	ATAATTACTATCAAATGCCCCAAC	CAAGATTTACCTCAAGAAATGGAT
	Ftind40	1502502-821	73	ATATGATTGCTCCAGTATTTATTTC	TTGTAAGGTGATCGGAGTATTT
	Ftind41	1494030-328	20	CCAAGAGCAGAGCATAATTCTAA	GCCTGACRCAATGACATATTTAC
	Ftind42	1849905-1850278	187	AGTAATAACGGTACGATCACAAAG	GGCTTTAGCTTACCAACASAAC
VNTR	Ft-M26	1833026-37	6‡	AATACTCGCTTCTATCTTTCTGGT	AATCTTTTGGAGAGGTTTTATTCA
SNP	Ft-SNP1	927939	1	ATCCCTGTTGGGATATCCTCGACTAA	ACCAAGGTAGATTTGCAGCTAC**A**§ ACCAAGGTAGATTTGCAGCTAC**G**§
	Ft-SNP2	1044580	1	atcagacttaggtgttagatcagagtt	tgaatactctacgcgataagat**a**§ tgaatactctacgcgataagat**g**§

*INDEL, insertion/deletion; VNTR, variable number tandem repeat; SNP, single nucleotide polymorphism. †GenBank accession no. AM233362.1 (complete genome of *F. tularensis* subsp. *holarctica* LVS). ‡There were two 6-bp repeats in the genome sequence of LVS. §The 2 SNP states are in **boldface**, an adenosine nucleotide represent a derived state for both markers.

### Genetic Groups, Subgroups, and Cluster Analysis

We assigned isolates to genotypes by using the binary character output for each INDEL marker, the repeat copy number at each VNTR marker, and the results of the SNP assay. We analyzed the associations among genotypes by their degree of genetic character sharing by using the eBURST algorithm (*17*). Genetic groups were defined as genotypes sharing 16 of 18 characters and subgroups sharing 17 of 18 characters. In addition, the genetic associations between the isolates were assessed by cluster analysis by using the unweighted pair group m with arithmetic mean (*18*) algorithm implemented in BioNumerics version 5.1 (Applied Maths NV, Sint-Martens-Latem, Belgium) using the categorical coefficient.

### Patient Interviews and Geographic Mapping

To obtain geographic data on likely places of disease transmission in Ljusdal, all patients in whom *F. tularensis* subsp. *holarctica* was diagnosed from 1995 through 2005 completed a questionnaire distributed during 2007 and marked on an accompanying map the location of the site where they believed they had been infected. In Örebro, patients admitted to hospital with tularemia from 2000 through 2004 were interviewed on admission, interviewed by telephone, or issued questionnaires, as previously described (*5*). All patients were asked for alternative places of disease transmission and to self-estimate the spatial data quality on a 3-category scale. A pair of RT-90 cartographic coordinates (*19*) was then assigned to each patient and the corresponding *F. tularensis* subsp. *holarctica* isolate, specifying the locations of first-choice place of transmission with the highest self-estimated quality. RT 90 is a standardized 2-dimensional Swedish map reference coordinate system. For patients who indicated multiple places of infection with identical data quality estimates, the coordinates of the place closest to the spatial mean center of tularemia in Ljusdal or Örebro were used (Appendix Table 1, and  Appendix Table 2). If no geographic information was available for a patient, the residential address was used.

We visualized and analyzed geographic and genotype data by using ArcView software in ArcGIS version 9.3 (ESRI, Redlands, CA, USA), and calculated spatial mean centers of disease occurrence (the average x and y values for the input coordinates) for all genetic groups and subgroups of isolates. The directional trend was examined by using the directional distribution tool in ArcGIS Spatial Statistics Tools (ESRI) and an ellipse size of 1 standard deviation.

## Results

### Descriptive Epidemiology

We isolated *F. tularensis* from 136 of 441 patients with laboratory-verified tularemia in the Municipality of Ljusdal during 1995–2005 (n = 56) and in the County of Örebro during 2000–2004 (n = 80). This finding constituted 34% (56/163) and 29% (80/278) of all patients with laboratory-verified tularemia during the outbreak years in Ljusdal (1995, 1998, 2002, 2005) and Örebro (2000, 2002–2004), respectively (Figure 1).

**Figure 2:**
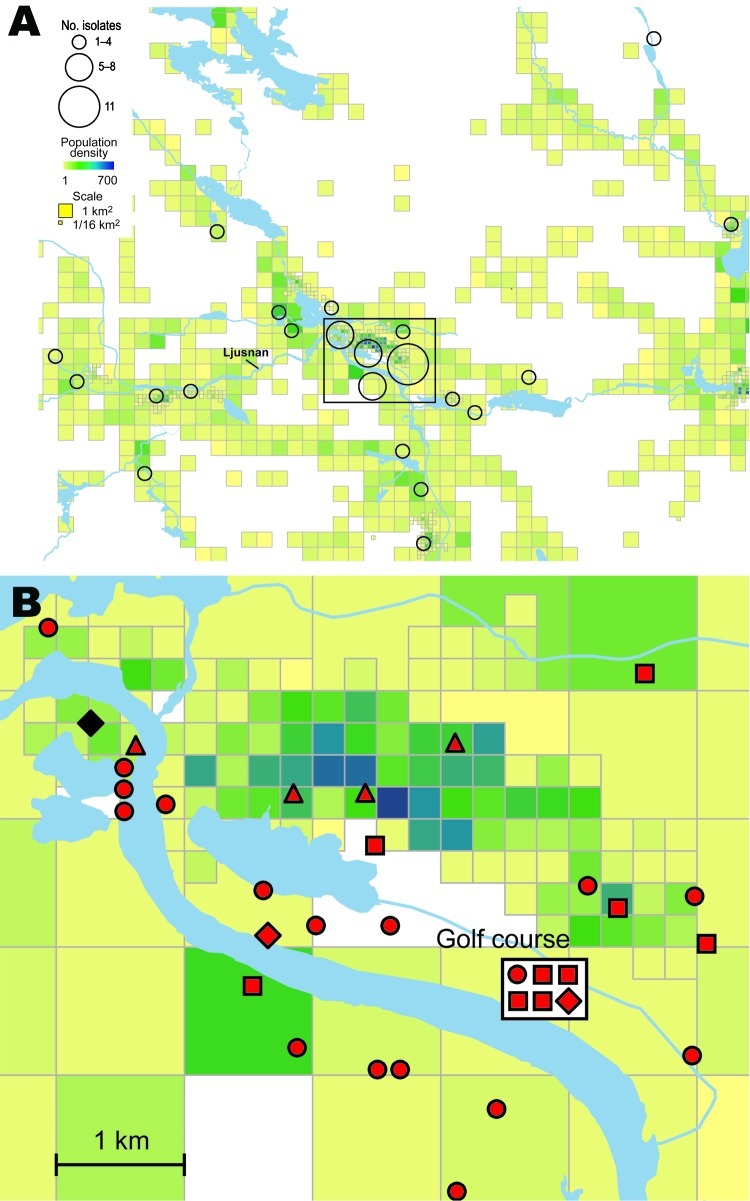
A) Spatial distribution of 56 places of tularemia transmission in Ljusdal, Sweden, 1995–2005, overlaid on a map with color-coded demographic data based on residential addresses. B) Disease cluster in an area of 25 km^2^ along the Ljusnan River in Ljusdal. Reported places of disease transmission and corresponding bacterial genotypes are shown. The 33 *Francisella tularensis* isolates belong to genetic group 1e and are of genotype ID 15 (red) or genotype ID 16 (black). Place of disease transmission was reported to be certain (circle), probable (square), or possible (diamond); patient residency was used when transmission data was unavailable (triangle).

**Figure 4:**
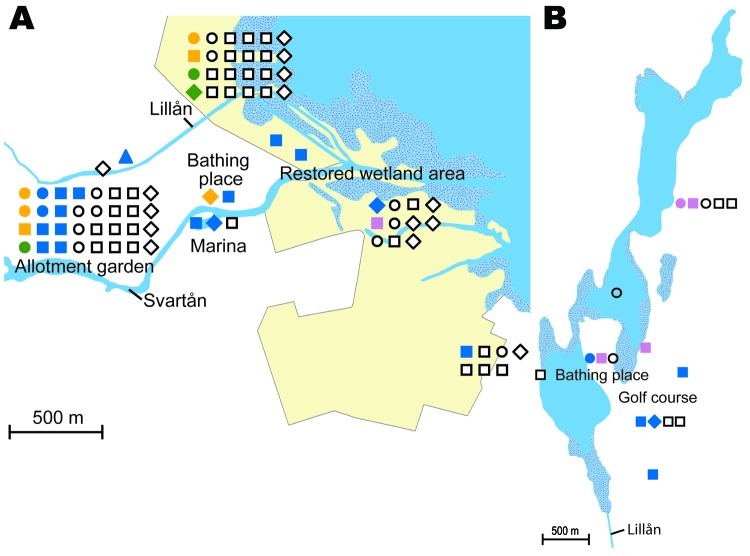
A) Cluster site for tularemia transmission at Oset/Rynningeviken nature reserve in Örebro, Sweden, with 83 patient reports. Twenty-seven *Francisella tularensis* isolates were recovered from these patients. B) Cluster site for tularemia transmission at Lake Lången, Örebro, Sweden, with 17 patient reports. Nine *F. tularensis* isolates were recovered from these patients. Place of disease transmission were reported to be certain (circle), probable (square), or possible (diamond); patient residency (triangle) was used if no such data was available. Genetic groups are indicated by color: yellow (1a), green (1b), blue (1d), or purple (2); white indicates no *F. tularensis* culture.

Analysis per outbreak year showed that the number of patients with culture-verified tularemia were proportional to the total number of tularemia patients. The ages of patients from whom positive cultures were obtained ranged from 1 to 90 years, distributed evenly among age groups: 20%, 18%, 34%, and 28% in persons 0–20, 21–40, 41–60, and >60 years of age, respectively, with a male:female ratio of 1.2:1 (Appendix Tables 1, 2). No apparent correlations were found between genotypes and either the gender or age of the patients. The annual incidence rates of tularemia were 21–423 per 100,000 persons in the Municipality of Ljusdal and 12–55 per 100,000 persons in the County of Örebro in outbreak years, but disease-free years were interspersed between outbreaks. Generally, tularemia patients reported that they were infected at places used for outdoor activities, e.g., on walking paths, at bathing places, at golf courses, or in an allotment garden (parcels of land for cultivation) assigned to individuals or families (Figures 2–4; Technical Appendix). A statement of a likely arthropod transmission vector was available for patients from Örebro; indicating mosquitoes (n = 101), ticks (*4*), horse flies (*8*), mosquitoes or ticks (*2*), mosquitoes or horse flies (*19*); 144 patients reported not knowing (the vectors reported by the 80 patients with culture-verified tularemia are listed in  Appendix Table 2). A presumed place of tularemia acquisition was pinpointed by 120 (88%) of 136 of patients and self-estimates of the quality of spatial data were available for 44 of 56 patients in Ljusdal and 76 of 80 patients in Örebro (Appendix Tables 1, 2) with culture-verified tularemia. Overall, patients felt confident about where they had acquired tularemia; 92 of 136 patients indicated that their place of disease acquisition was certain or probable (Table 2).

**Table 2:** Patient self-estimates of data quality for places of tularemia transmission, Sweden*

Location	Certain	Probable	Possible	Residential address	Total
Örebro	15	38	23	4	80
Ljusdal	23	16	5	12	56
Total	38	54	28	16	136

*Values summarize the information in the column “Patient self-estimate” found in Appendix Tables 1 and 2.

### Genetic Analysis of Outbreak Isolates

**Figure 5:**
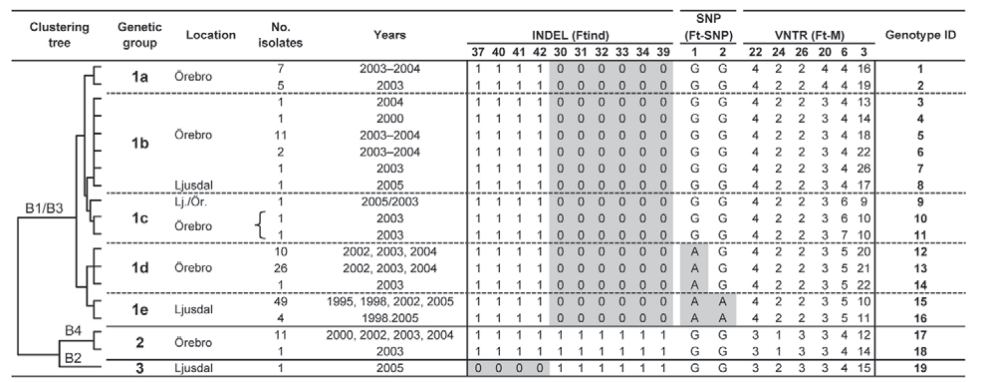
Attributes of 19 genotypes of *Francisella tularensis* subsp. *holarctica* identified in this study, and their genetic associations as assessed by a phylogenetic method (the clustering tree) or by an allele-based method (the genetic group designations). The letter and number designations in the clustering tree refer to nomenclatures of *F. tularensis* genetic clades as described by Johansson et al. (*9*). Gray shading indicates the derived genetic marker states. INDEL, insertion/deletion; SNP, single nucleotide polymorphism; VNTR, variable number of tandem repeats; ID, identification.

**Figure 6:**
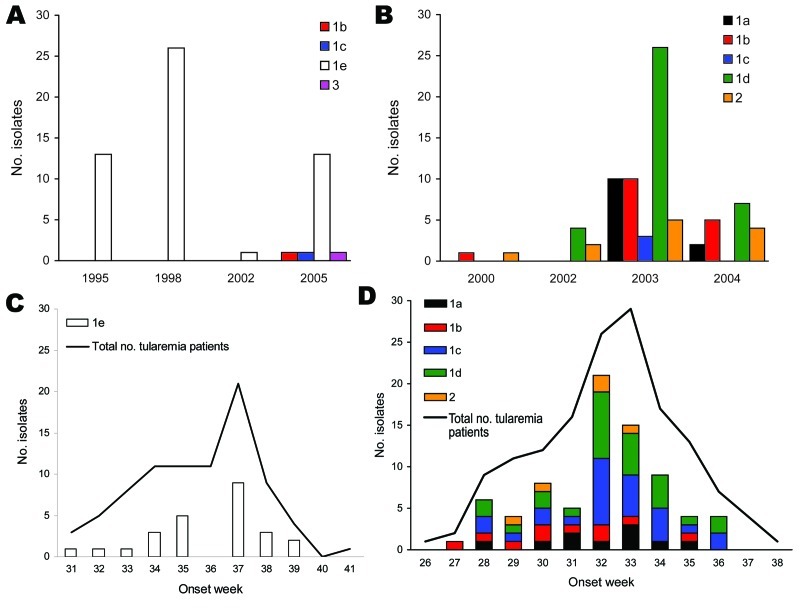
Proportional representation of genetic groups among isolated recovered per year for Ljusdal (A) and Örebro (B) and seasonal distribution of genetic groups of *Francisella tularensis* subsp. *holarctica* in 1998 in Ljusdal (C) and in 2003 in Örebro (D), Sweden. The week of disease onset was available for 84/87 patients in Ljusdal and 148/152 patients in Örebro.

We first identified 34 genetic markers that were polymorphic among *F. tularensis* subsp. *holarctica* isolates of worldwide origin. Among these, 18 markers (6/14 VNTRs, 10/18 INDELs, and both SNPs) were polymorphic among the 136 study isolates. Seven of these polymorphic markers have not been previously described (Table 1); 11 (Ftind30–34, Ftind37, Ft-M3, Ft-M6, Ft-M20, Ft-M22, Ft-M24) have been previously published (*9**,**15*). By applying the 18-marker system to the 136 isolates, we identified 19 genotypes. The 136 isolates were assigned to 3 genetic groups (denoted 1, 2, and 3) corresponding to previously described genetic groups (*9**,**15**,**20*) and 5 subgroups (denoted 1a–e), on the basis of their degree of character sharing (Figure 5). Most isolates from Ljusdal (55/56) and Örebro (68/80) belonged to genetic group 1. In pairwise comparisons, genetic groups 1, 2, and 3 differed at 6–13 of the 18 characters. A mutation at marker Ft-SNP2 was distinctive to the large genetic subgroup 1e from Ljusdal (53 isolates). Cluster analysis based on a distance matrix showed an identical grouping of isolates (Figure 5). Only 1 genotype was represented in both study locations; an isolate from Ljusdal obtained in 2005 was identical to an isolate from Örebro in 2003 (see genotype ID 9; Figure 5). In both study locations, genetic subgroups and genotypes were present throughout a tularemia season and persisted over years (Figures 5, 6).

### Phylogeographic Analysis in Ljusdal

**Figure 7:**
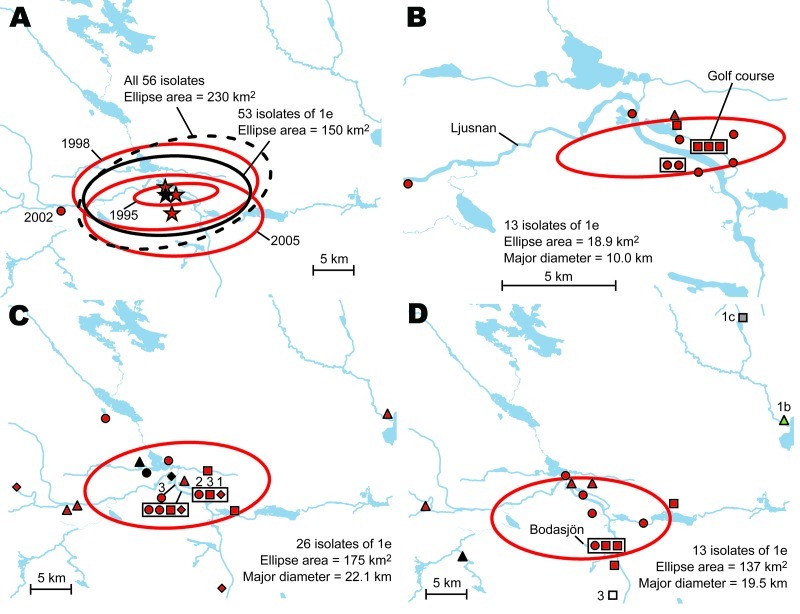
A) Directional distributions of tularemia transmission sites in Ljusdal, Sweden, by outbreak year (red ellipses). The *Francisella tularensis* isolates recovered from patients in Ljusdal were genetically monomorphic, with 53/56 isolates belonging to genetic subgroup 1e (solid black ellipse). The dashed black ellipse represents the distributions of all 56 isolates. Each ellipse represents a 1 standard deviation distribution around the mean centers of occurrence (starred). B) Distributions of 13 isolates of genetic group 1e, genotype identification (ID) 15 (red), Ljusdal, 1995. C) Distributions of 26 isolates of genetic group 1e, genotype ID 15 (red) and genotype ID 16 (black), Ljusdal, 1998. Numbers above symbols indicate multiple data points. D) Distributions of 13 isolates of genetic group 1e, genotype ID 15 (red) and genotype ID 16 (black); genetic group 1b (green); genetic group 1c (gray); and genetic group 3 (white), Ljusdal, 2005. Spatial data quality assessment for each pair of coordinates is shown as certain (circle), probable (square), or possible (diamond); patient residency (triangle) was used when transmission data were unavailable.

The *F. tularensis* isolates recovered from 1995 through 2005 from patients in Ljusdal were genetically monomorphic. The 53 of 56 isolates that belonged to genetic subgroup 1e were circumscribed by a 150-km^2^ ellipse (Figure 7, panel A). All 56 isolates were circumscribed by a 230-km^2^ ellipse. Analysis per outbreak year showed that the infection area of 1e isolates was stable, with an east–west distribution along the river Ljusnan (Figure 7). The places of disease transmission for 3 isolates of genetic subgroups 1b, 1c, and 3 were peripheral to the infection area of 1e isolates (Figure 7, panel D). Many patients reported acquiring tularemia from restricted geographic areas, e.g., 33 of 56 isolates were from a 25-km^2^ stretch along the river Ljusnan, with a disease cluster at a golf course (Figure 2).

### Phylogeographic Analysis in Örebro

**Figure 8:**
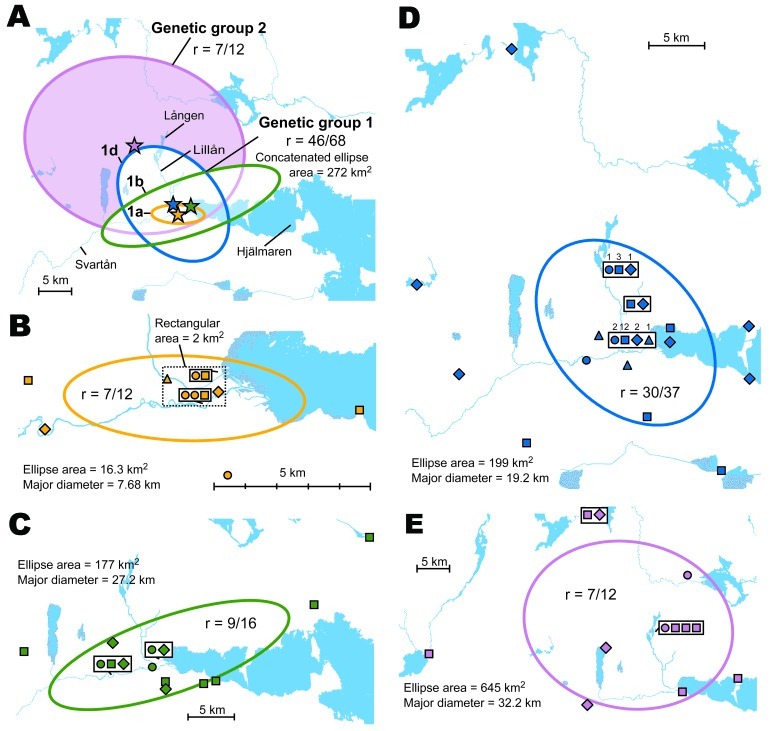
A) Directional distributions and spatial mean centers for 80 *Francisella tularensis* isolates of 4 different genetic groups, Örebro, Sweden. Each colored ellipse represents a 1 standard deviation distribution around the mean centers of occurrence (starred) for a genetic group. B–E) Details on transmission sites in Örebro for genetic groups of *F. tularensis* isolates: B) genetic group 1a; C) genetic group 1b; D) genetic group 1d; E) genetic group 2. Patient self-estimates of the spatial data quality are shown as certain (circle), probable (square), or possible (diamond); patient residency (triangle) was used if no such data were available. Proportions (r) of transmission sites within/outside an ellipse are indicated. Numbers above symbols in panel D indicate multiple data points at the same place.

In Örebro, the places of disease transmission for genetic groups 1 and 2 were within 2 partially overlapping geographic areas with distinctly separate mean centers of occurrence (Figure 8, panel A). Closer examination of the areas of genetic group 1 showed that those of subgroups 1a, 1b, and 1d had similar spatial centers but different directional distributions (Figure 8, panels B–D). Infection locations of genetic subgroups 1a and 1b were oriented in an east-west direction along the Svartån River, whereas those of subgroup 1d were oriented in a north-south direction along the Lillån River. The places of disease transmission of genetic group 1 were circumscribed by a concatenated 272-km^2^ ellipse area (Figure 8, panel A); those of genetic group 2 were circumscribed by a 645-km^2^ ellipse (Figure 8, panel E). The proportions of isolates transmitted within the elliptic infection areas of genetic subgroups 1a, 1b, 1d, and 2 were 7 of 12, 9 of 16, 30 of 37, and 7 of 12, respectively (Figure 8, panels B–E). The geographic distribution of genetic subgroup 1a’s places of transmission was most restricted, spanning an elliptic area of only 16 km^2^. Seven of 12 transmission locations of genetic subgroup 1a could alternatively be enclosed in a rectangular area of just 2 km^2^ (Figure 8, panel B). The genetic subgroup 1c comprised only 3 isolates with 3 distinct genotypes; places of disease transmission spanned a distance of 58 km (not shown).

**Figure 3:**
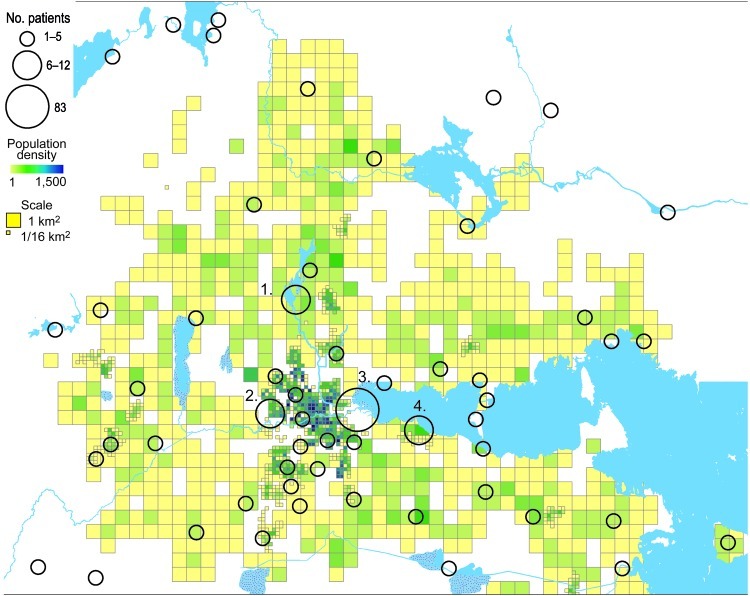
Geographic distribution of 202/240 places of tularemia transmission in Örebro, Sweden, 2000–2004. Four recreational areas were disease cluster sites for tularemia transmission: 1) Lake Lången, 2) Karslundsskogen/Hästhagen, 3) Oset/Rynningevikens nature reserve, and 4) Ekeby-Almby.

In total, 240 of 278 patients in Örebro reported places of tularemia transmission. A spatial distribution plot of the locations reported by 202 of these patients is shown in Figure 3 (those of 38 patients are not shown because they were outside the map area), indicating the existence of 4 tularemia transmission clusters. Two clusters are further detailed in Figure 4. Eighty-three patients reportedly acquired tularemia in the Oset/Rynningeviken nature reserve 2 km from Örebro City center. Twenty-six of 27 cultured *F. tularensis* isolates from this area belonged to genetic group 1. Seventeen patients reported transmission at Lake Lången, 5 km north of Örebro City. Here, multiple patient reports verified that bacteria of genetic groups 1d and 2 coexisted. However, the centers of occurrence were geographically distinct (Figure 8, panel A).

## Discussion

In this study of natural outbreaks of human tularemia in 2 locations in Sweden, genetic techniques developed for high-resolution typing of *F. tularensis* were used in conjunction with information obtained from patient surveys and interviews to investigate the epidemiology and geographic spread of disease. Places of transmission of specific *F. tularensis* genotypes (Figure 5) were highly localized and restricted to areas as low as 2 km^2^, pinpointing likely point sources of infection (Figures 7, 8). The results demonstrate the capability of enhancing epidemiologic investigations of tularemia by combining data from patient interviews with high-resolution genotyping of *F. tularensis* isolates recovered from the same patients.

A recent study in Utah of 5 patients and 11 rabbit carcasses infected with *F. tularensis* indicated that multiple *F. tularensis* subspecies and genetic subgroups may cause tularemia in a localized outbreak (*21*). Other studies have demonstrated that genetic subgroups of *F. tularensis* have distinct frequencies at continental scales throughout the Northern Hemisphere (*20**,**22**–**24*). Our study, in which 136 *F. tularensis* subsp. *holarctica* isolates from 2 localized human outbreaks were examined, shows the phylogeographic structure in *F. tularensis* subsp. *holarctica* populations involved in local outbreaks.

Why is there a phylogeographic structure? We found high genetic diversity and limited spatial distribution of genetic group 1 isolates in Örebro, which suggested recent expansion of local *F. tularensis* subsp. *holarctica* populations. The number of genotypes in genetic group 1 was notably greater than among genetic group 2 isolates from Örebro or among all the isolates recovered in the long-term tularemia-endemic area of Ljusdal (Figure 5). Because previous genotyping data have demonstrated that homoplastic SNP mutations are virtually nonexistent in *F. tularensis* (*20**,**25*), a common mutation at Ft-SNP1 (Figure 5) indicates that the isolates in genetic subgroup 1d in Örebro and 1e in Ljusdal share a more recent common ancestor than they do with isolates of subgroups 1a, 1b, or 1c in Örebro. However, genetic distances among all the group 1 isolates are likely to be small because Ft-SNP1 could be identified only by comparison of complete genome sequences, including a genetic subgroup 1e genome (strain FSC200 from Ljusdal) (*26**,**27*). Altogether, the data imply that genetic groups 1a, 1b, and 1c isolates have a local evolutionary history rather than a recent local disease introduction (as verified by high genetic variation at VNTR markers) and that the 1d isolates appear genetically related with isolates from Ljusdal (as verified by a SNP mutation).

Restoration of a wetland area between 1993 and 2006 (Figure 4) in Örebro may have been a factor in expansion of these genetic subgroups. Because *F. tularensis* subsp. *holarctica* is known to be associated with natural waters, favorable conditions for its replication may have resulted. The large genetic distance between genetic groups 1 and 2 in Örebro (they are consistently distinct at 9 of 18 markers; Figure 5), where tularemia recently has reemerged, compares with distances previously found among *F. tularensis* subsp. *holarctica* isolates of worldwide origin (*9*) and excludes a recent local common origin. The existence of several distinct *F. tularensis* populations active within a single tularemia outbreak is further demonstrated by comparing data of this study with data from previous work by Johansson et al. (*9*) and Kugeler et al. (*24*). All of the group 1 isolates belong to clades named B1/B3 or B.Br.013/014, respectively in these previous publications; group 2 isolates belong to clade B4 or B.Br.007/008 (or a nearby clade), and group 3 isolates to clade B2 or B.Br.OSU18. Kugeler et al. demonstrated the large numbers of SNPs separating group 1 from groups 2 and 3 isolates, thus verifying very distinct genetic populations.

The geographically widely distributed genetic group 2 in Örebro, the subgroup 1d in Örebro, or the subgroup 1e in Ljusdal, may be results of past temporary reductions of population sizes (genetic bottlenecks) or selective events (selective sweeps) that have eliminated genetic variation in originally more diverse populations. The selective sweep hypothesis is particularly attractive; a highly fit genotype over time will increase its frequency relative to other genotypes and may occupy larger geographic areas. This scenario would explain previous findings of low genetic diversity among isolates recovered from areas of Sweden with a long history of tularemia infections (*9*).

The genetic differences of genetic groups and subgroups were mirrored spatially. The genetic groups 1 and 2 in Örebro showed distinct mean centers of occurrence (Figure 8, panel A), and genetic group 1d isolates were the only isolates of group 1 found along the whole stretch of the river Lillån, resulting in a distribution area oriented north-south, opposite to subgroups 1a and 1b, which showed an east-west direction of their distribution areas (Figure 8, panels B–D). Similar pattern of separated disease occurrence center for genetic groups were found in Ljusdal (Figure 7, panel D). Collectively, these observations indicate distinct replication foci and dispersal areas of different *F. tularensis* subsp. *holarctica* populations.

Many reasons are likely for a clustering of human tularemia. First, tularemia occurrence depends on the number of persons at risk, i.e., those who visit or live in areas where *F. tularensis* foci exists (Figures 2, 3). Second is the effect of vector ecology. Most of the 134 (91%) patients in Örebro who specified a disease transmission vector reported it to be mosquitoes. Thus, the distance that mosquitoes disperse, 200–2,000 m for most species in Sweden (*28**,**29*), probably strongly influences the infection patterns. Third, local factors affect the persistence and distribution of *F. tularensis* in nature. We found identical genotypes over different years, indicating that tularemia overwinters at the disease cluster sites. Genetic groups also were present during the whole tularemia season from July to September, indicating that no particular temporal patterns were associated with specific bacterial genotypes (Figures 5, 6).

Although uneven distributions of persons at risk and transmission vectors, as well as a general association of tularemia with streaming waters, may explain geographic disease clustering in humans, only different spatial distributions of *F. tularensis* populations can explain clustering of genetic groups and subgroups. Our observations are consistent with Pavlovsky’s theory of “natural nidality of transmissible diseases,” i.e., a connection of the vector-borne disease with a definite geographic landscape (*30*). In the case of tularemia, a recent study on dog ticks carrying *F. tularensis* subsp. *tularensis* on Martha’s Vineyard (Massachusetts, USA) showed persistence in a microfocus in nature over 4 years (*31*). Supporting the existence of a landscape epidemiology of tularemia, we found that different *F. tularensis* subsp. *holarctica* populations are locally present near certain bodies of water where they apparently stably perpetuate.

A major limitation of this study is a retrospective design that may have caused recall bias regarding the locations at which tularemia was contracted. The patient recall time in Ljusdal sometimes was up to 12 years; in Örebro patients were approached at admission to hospital. It is our impression, however, from many patient interviews, that the short incubation time of tularemia (2–5 days), the distinct clinical expression, its occurrence in restricted geographic areas, and a transmission route by blood-feeding arthropods, did facilitate patient recalls.

Ljusdal and Örebro in Sweden have comparatively high tularemia incidence rates. The results of this study suggest that genotyping coupled with global imaging satellite mapping can help identify local environmental sources of tularemia, which is essential for effective infection control. This study also shows that pathogen genome sequencing efforts can contribute to the design of genotyping schemes tailored to a specific outbreak investigation. By combining high-resolution genotyping with patient interviews, we found *F. tularensis* populations to have strong spatial associations in 2 localized tularemia outbreaks. In future investigations, we believe that application of parallel mass-sequencing technologies to *F. tularensis* will be highly valuable for identifying additional genetic markers that, in turn, will facilitate tracking of the zoonotic pathogen through environmental sources, blood-feeding arthropods, and mammals. In addition to a more detailed genetic analysis, we need to identify ecologic correlates to the local areas of *F. tularensis* persistence and replication. Ultimately, the goal is to gain knowledge enabling future focused interventions directed at reducing the risk for tularemia acquisition by humans visiting or living in areas in which tularemia is highly endemic.

## Supplementary Material

Appendix Table 1 Isolate and patient information, Francisella tularensis infections, Ljusdal, Sweden

Appendix Table 2 Isolate and patient information, Francisella tularensis infections, Örebro, Sweden

Technical Appendix Landscape Epidemiology of Tularemia Outbreaks in Sweden
