# Supplementary material for: Landscape Epidemiology of Tularemia Outbreaks in Sweden
Source: Emerg Infect Dis. 2009 Dec;15(12):1937–47. doi: 10.3201/eid1512.090487 (PMC3044527; doi:10.3201/eid1512.090487)
Supplement: Appendix Table 1 — Isolate and patient information, Francisella tularensis infections, Ljusdal, Sweden [file 09-0487_appT1-s1.pdf]

Publisher: CDC; Journal: Emerging Infectious Diseases
Article Type: Research; Volume: 15; Issue: 12; Year: 2009; Article ID: 09-0487
DOI: 10.3201/eid1512.090487; TOC Head: Research

Appendix Table 1. Isolate and patient information, *Francisella tularensis* infections, Ljusdal, Sweden

Year	Onset week	Genetic group	Genotype identification	Age, y/sex	Patient self-estimate*	FSC no.†
1995	32	1e	15	13/M	1	173
		1e	15	22/F	2	174
		1e	15	32/F	1	325
		1e	15	13/F	1	326
	33	1e	15	52/M	1	328
		1e	15	12/F	2	329
		1e	15	50/M	2	179
		1e	15	47/F	1	244
		1e	15	25/M	4	245
		1e	15	4/M	1	330
	34	1e	15	15/F	1	165
		1e	15	37/F	2	167
	35	1e	15	64/F	1	171
1998	31	1e	15	4/F	2‡	200§
	32	1e	15	4/M	2‡	201
	33	1e	15	7/M	1	202
	34	1e	15	53/F	1	204
		1e	15	51/F	1	203
		1e	15	21/M	3	205
	35	1e	15	36/M	3	206
		1e	16	31/M	4	207
		1e	16	54/F	1	208
		1e	15	54/F	2	209
		1e	15	52/F	1	210
	37	1e	16	48/M	3‡¶	211
		1e	15	4/M	4	212
		1e	15	6/F	2‡	213
		1e	15	10/F	2‡	218
		1e	15	59/M	1	226
		1e	15	83/M	4	214
		1e	15	68/M	4	215
		1e	15	34/F	1	216
		1e	15	34/M	3	217
		1e	15	78/F	3	219
	38	1e	15	59/F	1	224
		1e	15	1/F	1‡	222
		1e	15	66/M	2	225
	39	1e	15	27/M	4	223
		1e	15	38/F	1	227
2002	33	1e	15	31/F	4	346
2005	28	1e	15	9/M	4	626
	29	1e	15	44/M	2‡	627
	31	1e	15	58/M	1	629
		1e	15	11/M	4	630
		1e	15	69/M	2	631
	32	1e	15	41/F	1	636
		1e	15	69/M	1	645
	33	1e	15	53/F	2‡	634
	34	1c	9	52/F	2‡	638
		3	19	44/M	2‡	641
		1e	15	90/M	1	648
	35	1e	15	36/M	4	646
		1e	15	12/M	1	649
		1e	15	58/M	2	650
	39	1b	8	59/M	4	661
		1e	16	13/F	4	662

*Patient self-estimate of spatial data quality: 1, certain; 2, probable; 3, possible; 4, only residential address was available.
†FSC, *Francisella* Strain Collection (FOI, Umeå, Sweden).

‡The patient indicated multiple places of infection.
§A draft genome sequence of isolate FSC200 is available at GenBank with accession no. AASP00000000.
¶The patient indicated multiple places of infection with identical data quality estimates.
