# Supplementary material for: Landscape Epidemiology of Tularemia Outbreaks in Sweden
Source: Emerg Infect Dis. 2009 Dec;15(12):1937–47. doi: 10.3201/eid1512.090487 (PMC3044527; doi:10.3201/eid1512.090487)
Supplement: Appendix Table 2 — Isolate and patient information, Francisella tularensis infections, Örebro, Sweden [file 09-0487_appT2-s2.pdf]

Publisher: CDC; Journal: Emerging Infectious Diseases
Article Type: Research; Volume: 15; Issue: 12; Year: 2009; Article ID: 09-0487
DOI: 10.3201/eid1512.090487; TOC Head: Research

Appendix Table 2. Isolate and patient information, *Francisella tularensis* infections, Örebro, Sweden

| Year | Onset week | Genetic group | Genotype identification | Age, y/sex | Vector* | Patient self-estimate† | FSC no.‡ |
|---|---|---|---|---|---|---|---|
| 2000 | 33 | 2 | 17 | 74/F | – | 1 | 285 |
| | 37 | 1b | 4 | 11/M | m | 2 | 279 |
| 2002 | 33 | 1d | 12 | 59/F | – | 2§ | 348 |
| | | 2 | 17 | 40/F | m | 2§ | 345 |
| | 35 | 2 | 17 | 67/F | m | 2 | 347 |
| | | 1d | 13 | 45/M | – | 3§¶ | 343 |
| | 37 | 1d | 13 | 10/M | m | 2§ | 341 |
| | 38 | 1d | 13 | 60/M | – | 2 | 351 |
| 2003 | 27 | 1b | 5 | 84/M | t | 1 | 371 |
| | 28 | 1d | 13 | 3/M | m | 1 | 362 |
| | | 1b | 5 | 37/F | h,m | 2 | 369 |
| | | 1d | 13 | 64/M | – | 3 | 432 |
| | | 1a | 2 | 53/M | m | 1 | 401 |
| | 29 | 1c | 10 | 75/M | h,m | 1 | 364 |
| | | 2 | 18 | 64/M | – | 2 | 366 |
| | | 1b | 5 | 5/M | t | 2§ | 363 |
| | | 1d | 13 | 58/F | m | 2 | 361 |
| | 30 | 1d | 13 | 61/F | m | 1 | 377 |
| | | 2 | 17 | 40/M | – | 3 | 397 |
| | | 1a | 2 | 68/F | m | 2 | 396 |
| | | 1d | 13 | 69/M | – | 2§ | 398 |
| | | 1b | 5 | 16/M | – | 3§¶ | 399 |
| | | 1b | 5 | 52/M | m | 2 | 400 |
| | 31 | 1d | 13 | 65/F | h,m | 3§¶ | 380 |
| | | 1b | 6 | 16/F | – | 3§¶ | 391 |
| | | 1c | 9 | 57/F | m | 1 | 378 |
| | | 1a | 2 | 17/F | – | 3§¶ | 379 |
| | | 1a | 1 | 45/M | – | 2§ | 392 |
| | 32 | 2 | 17 | 42/M | – | 3§¶ | 376 |
| | | 1d | 13 | 70/F | m | 2 | 389 |
| | | 1b | 5 | 65/M | m | 3 | 430 |
| | | 1d | 13 | 30/M | m | 2§ | 393 |
| | | 1d | 12 | 37/M | – | 3§¶ | 394 |
| | | 1d | 13 | 14/M | – | 4 | 421 |
| | | 1d | 13 | 3/F | – | 2 | 372 |
| | | 1c | 11 | 76/M | – | 2 | 408 |
| | | 1d | 13 | 11/M | – | 4 | 424 |
| | | 1d | 13 | 69/M | h,m | 2§ | 406 |
| | | 1b | 5 | 49/M | – | 3§¶ | 405 |
| | | 1a | 1 | 47/F | m | 2§ | 418 |
| | | 2 | 17 | 53/M | m | 3§¶ | 388 |
| | | 1d | 13 | 54/F | – | 1 | 420 |
| | 33 | 1a | 1 | 43/M | h,m | 3§¶ | 384 |
| | | 1d | 13 | 37/M | m | 3§¶ | 449 |
| | | 1d | 13 | 51/M | m | 3 | 419 |
| | | 1a | 2 | 25/M | m | 3§¶ | 423 |
| | | 1d | 13 | 27/F | – | 3 | 443 |
| | | 2 | 17 | 75/M | m | 1 | 426 |
| | | 1d | 13 | 63/M | m | 2§ | 410 |
| | | 1a | 2 | 36/M | m | 2 | 416 |
| | | 1b | 7 | 73/F | m | 1 | 425 |
| | | 1d | 13 | 35/F | m | 2 | 412 |
| | 34 | 1d | 12 | 57/M | – | 2 | 409 |
| | | 1d | 14 | 55/M | m | 2§ | 429 |
| | | 1a | 1 | 79/F | m | 1 | 434 |
| | | 1d | 12 | 52/M | – | 3§¶ | 438 |

| | | | | | | | |
|---|---|---|---|---|---|---|---|
| | | 1d | 13 | 66/F | – | 3§¶ | 442 |
| | 35 | 1b | 5 | 52/F | h | 1 | 440 |
| | | 1d | 12 | 43/M | – | 4 | 446 |
| | | 1a | 1 | 39/F | m | 1 | 444 |
| | 36 | 1d | 12 | 83/F | m | 1 | 447 |
| | | 1d | 12 | 51/M | m | 3§¶ | 448 |
| 2004 | 31 | 2 | 17 | 52/F | h,m | 2 | 519 |
| | 32 | 1a | 1 | 67/F | h | 1 | 521 |
| | 33 | 1b | 5 | 63/F | m | 2 | 523 |
| | | 1a | 1 | 74/F | – | 4 | 527 |
| | | 1d | 13 | 71/F | m | 2§ | 538 |
| | | 2 | 17 | 36/F | m | 2 | 535 |
| | 34 | 2 | 17 | 69/F | – | 2 | 529 |
| | | 1b | 6 | 47/M | – | 3 | 532 |
| | | 1d | 12 | 69/F | – | 2 | 542 |
| | | 1b | 5 | 57/F | m | 2 | 526 |
| | | 2 | 17 | 12/M | – | 2 | 540 |
| | | 1d | 13 | 81/M | m | 2 | 531 |
| | | 1d | 12 | 62/F | m | 2 | 534 |
| | 35 | 1d | 13 | 55/M | m | 2§ | 533 |
| | | 1b | 3 | 56/F | h,m | 2 | 548 |
| | 36 | 1b | 5 | 72/M | – | 3§¶ | 546 |
| | | 1d | 12 | 66/F | m | 2 | 547 |
| | 38 | 1d | 13 | 55/M | – | 2 | 549 |

*Tularemia transmission vectors pinpointed by the patient were mosquitoes (m), ticks (t), horse flies (h), and unknown (–).
†Patient self-estimate of spatial data quality: 1, certain; 2, probable; 3, possible; 4, only residential address was available.
‡FSC, *Francisella* Strain Collection (FOI, Umeå, Sweden).
§The patient indicated multiple places of infection.
¶The patient indicated multiple places of infection with identical data quality estimates.
