# Supplementary material for: Landscape Epidemiology of Tularemia Outbreaks in Sweden
Source: Emerg Infect Dis. 2009 Dec;15(12):1937–47. doi: 10.3201/eid1512.090487 (PMC3044527; doi:10.3201/eid1512.090487)

Landscape Epidemiology of Tularemia Outbreaks in Sweden

Technical Appendix

Satellite overviews and photos of tularemia foci in Ljusdal and Örebro, Sweden, to illustrate the local environmental conditions.

Appendix Figures 1-2 correspond to Figure 2 in article.

Figure 1. Central parts of Ljusdal municipality. **© Lantmäteriet Gävle 2009. Grant I 2009/0301.**

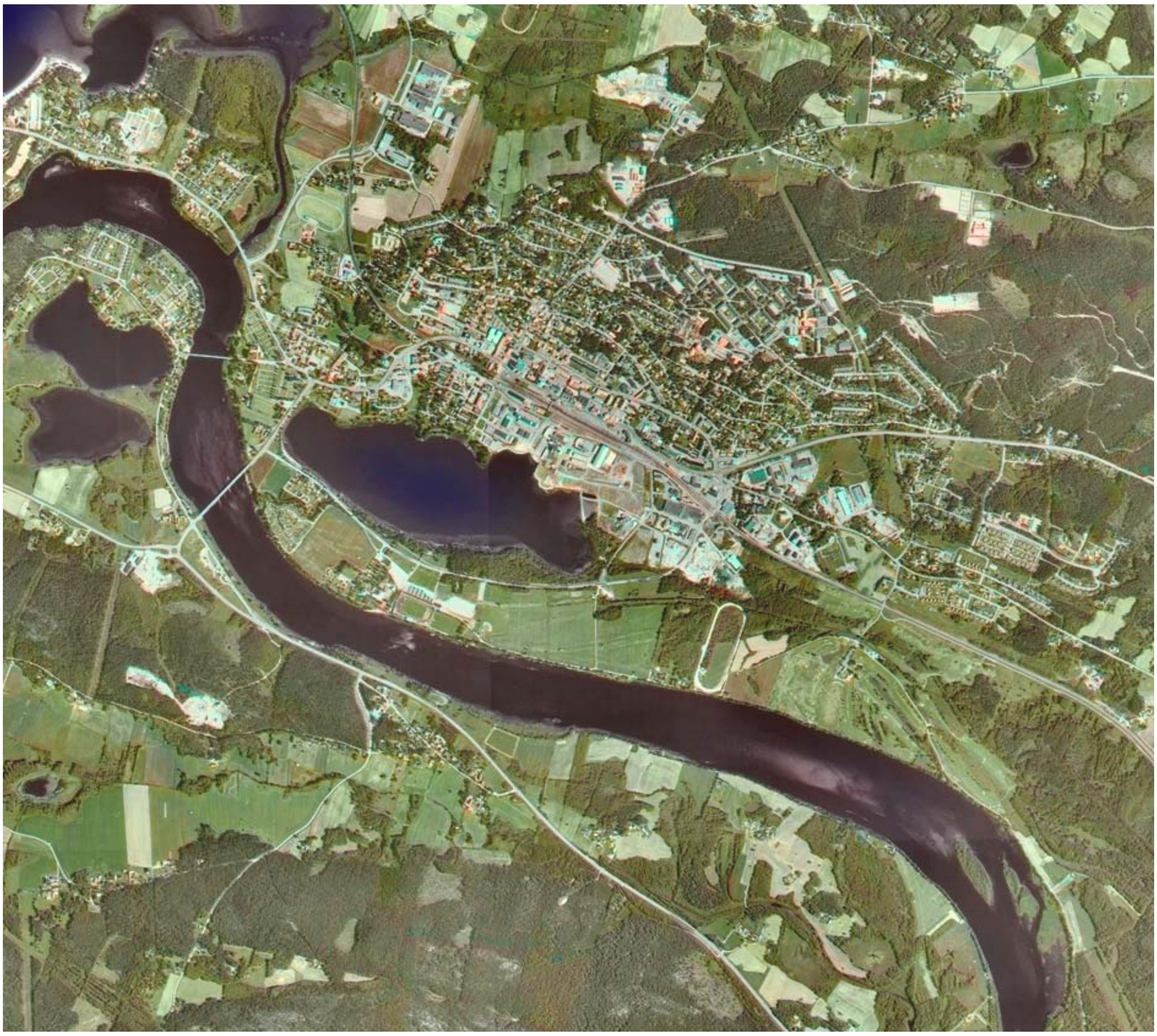

Figure 2. The golf course in Ljusdal.

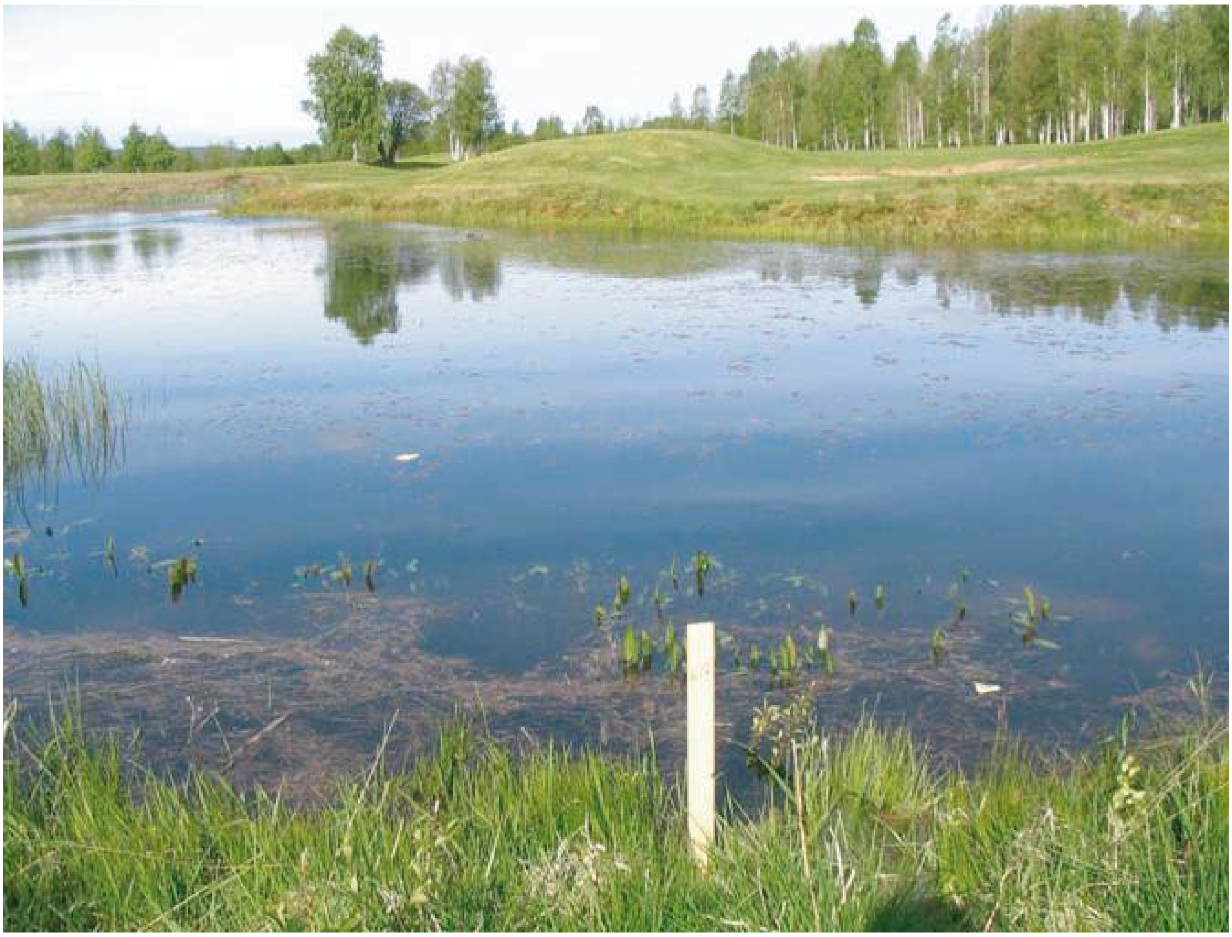

Appendix Figures 3-6 correspond to Figure 4 in article.

Figure 3. Eastern parts of Örebro city and the Oset/Rynningeviken nature reserve. **a)** Oset/Rynningeviken waterpark recreational area in Örebro, a restored wetland area established between 1993 and 2006 on a former waste disposal site. **b)** The Örebro marina and Alnängarna bathing place. **c)** Alnängarna allotment garden in central parts of Örebro with approximately 300 cottages. © **Lantmäteriet Gävle 2009. Grant I 2009/0301.**

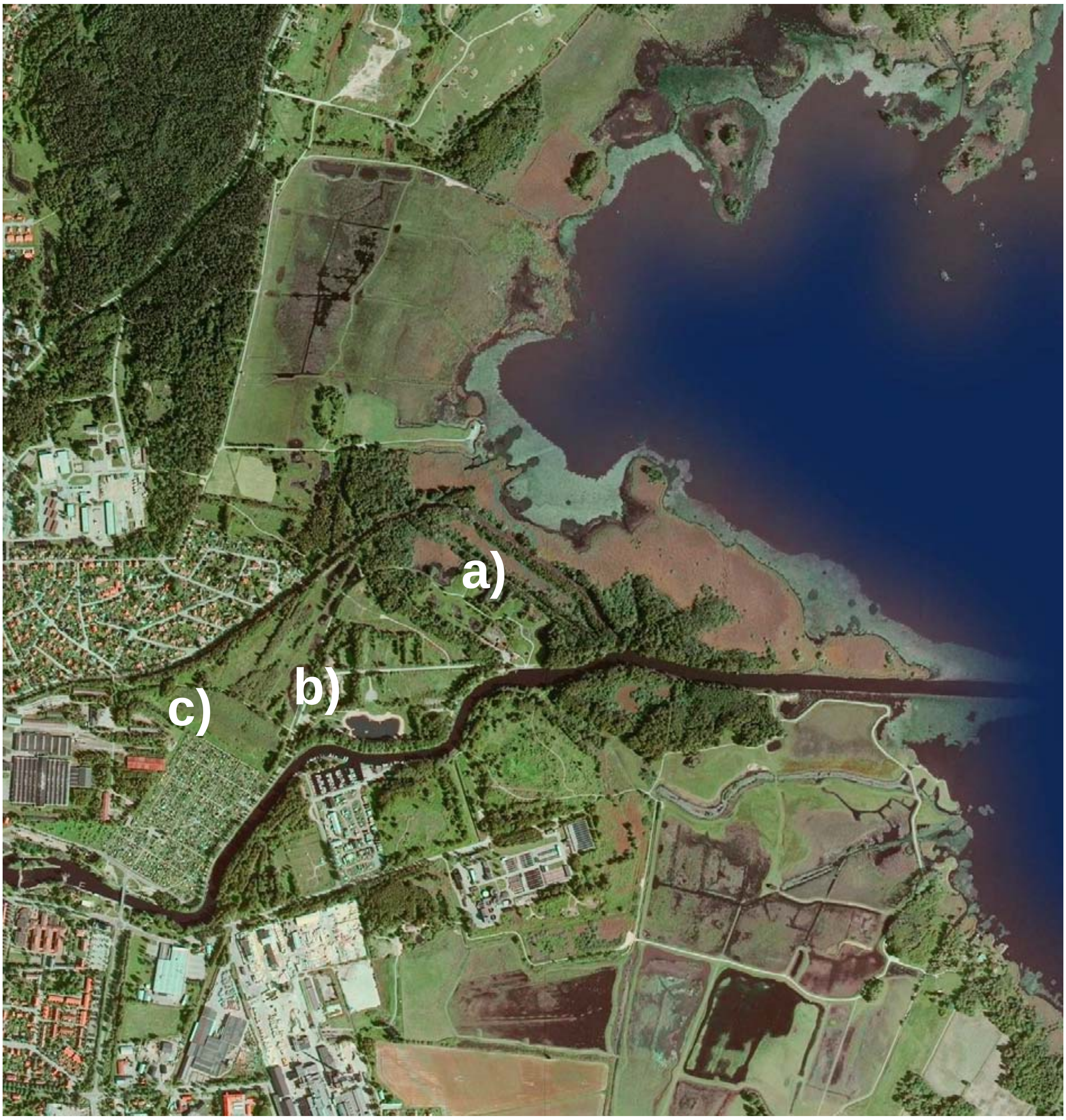

Figure 4. The Örebro marina and Alnängarna bathing place. © **Lantmäteriet Gävle 2009. Grant I 2009/0301.**

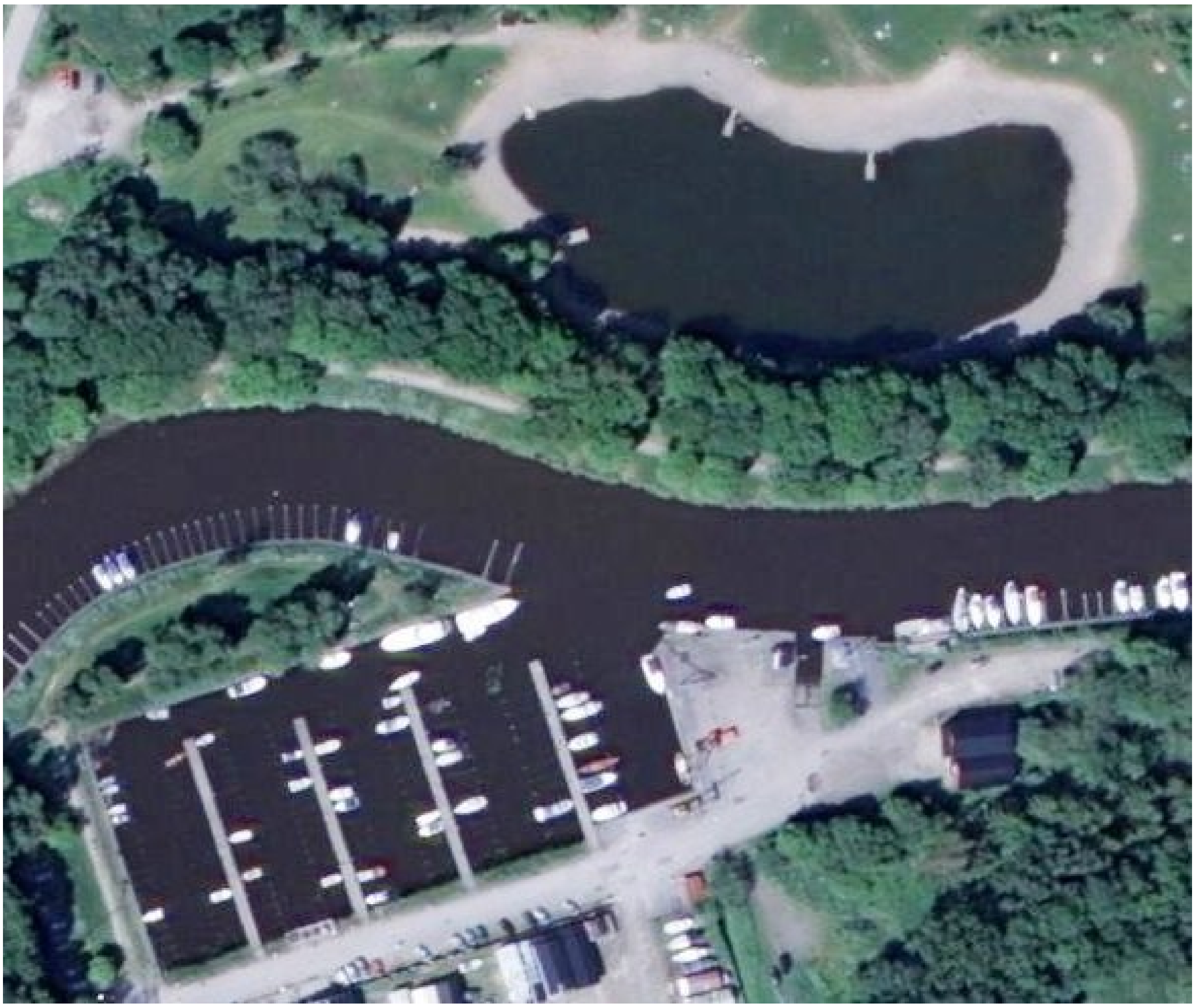

Figure 5. Alnängarna allotment garden in central parts of Örebro with approximately 300 cottages.

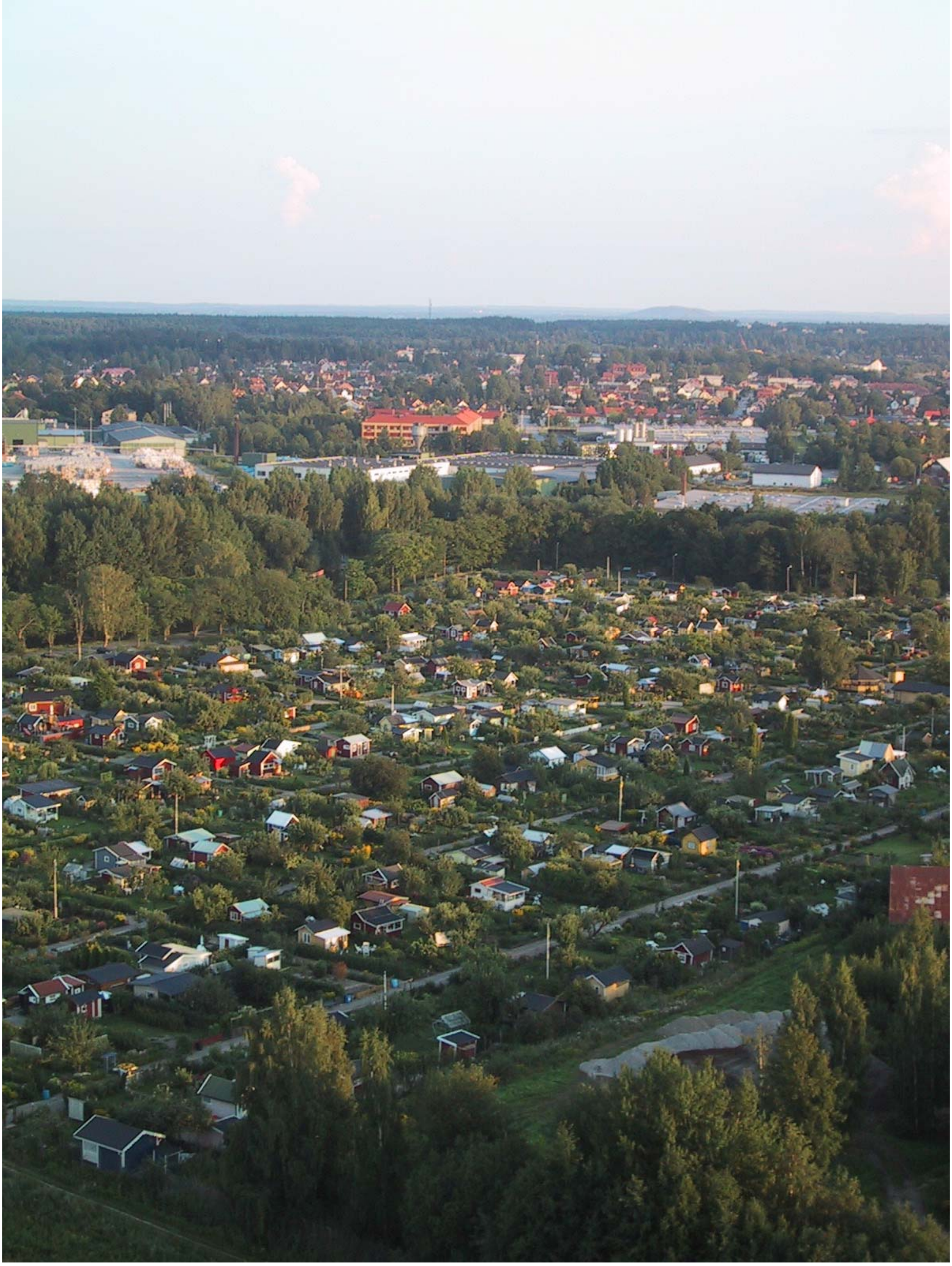

Figure 6. Lake Lången, 5 km N. Örebro. © **Lantmäteriet Gävle 2009. Grant I 2009/0301.**

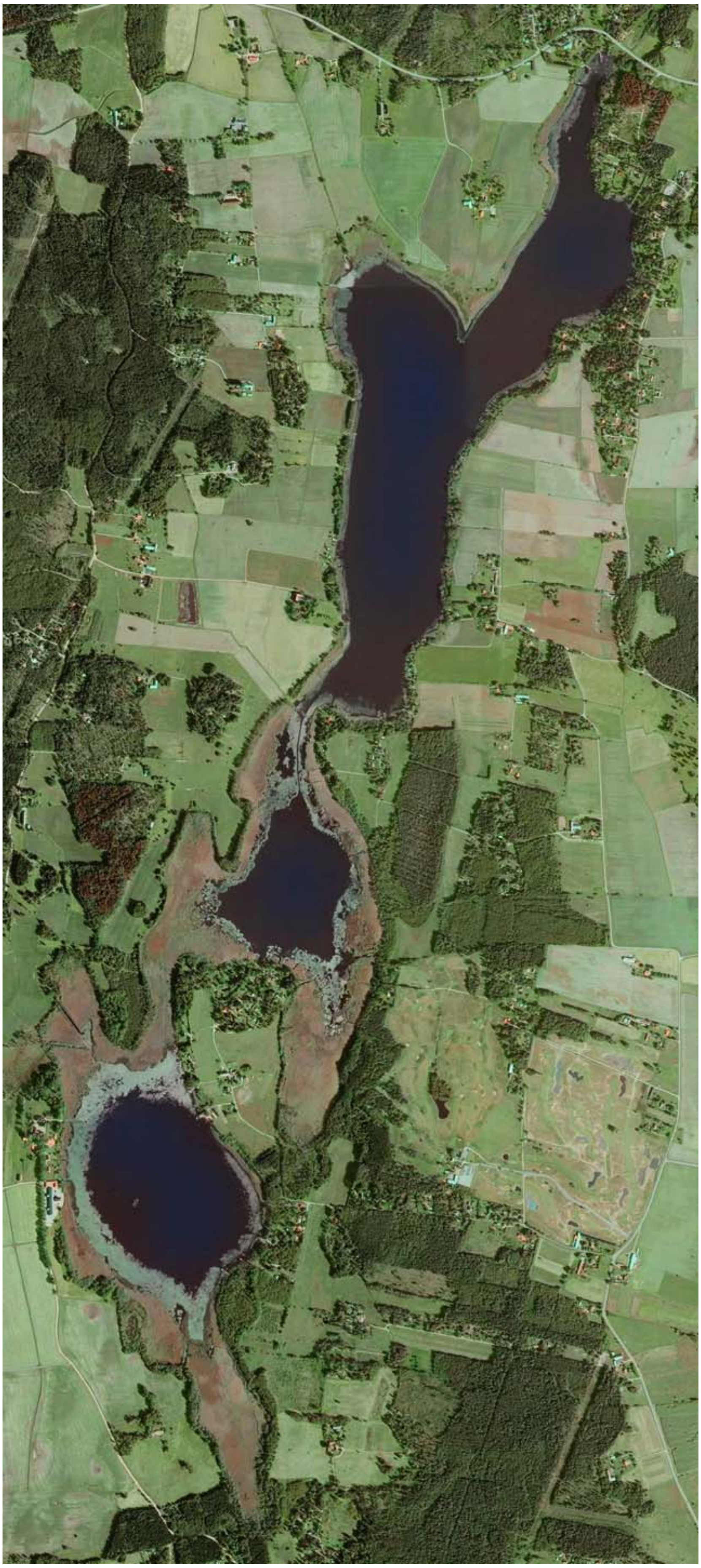

Supplement: Technical Appendix — Landscape Epidemiology of Tularemia Outbreaks in Sweden [file 09-0487_Techapp-s3.pdf]
